# Using PM2.5 concentrations to estimate the health burden from solid fuel combustion, with application to Irish and Scottish homes

**DOI:** 10.1186/1476-069X-12-50

**Published:** 2013-06-19

**Authors:** Karen S Galea, J Fintan Hurley, Hilary Cowie, Amy L Shafrir, Araceli Sánchez Jiménez, Sean Semple, Jon G Ayres, Marie Coggins

**Affiliations:** 1 Centre for Human Exposure Science, Institute of Occupational Medicine (IOM), Edinburgh, UK; 2 Institute of Occupational Medicine (IOM), Edinburgh, UK; 3 Harvard School of Public Health, Harvard University, Boston, MA, USA; 4 Division of Applied Health Sciences, University of Aberdeen, Aberdeen, UK; 5 Institute of Occupational and Environmental Medicine, University of Birmingham, Birmingham, UK; 6 School of Physics, National University of Ireland, University Road, Galway, Ireland

**Keywords:** Health burden assessment, Solid fuels, Peat, Scotland, Ireland, PM_2.5_

## Abstract

**Background:**

This study estimates the potential population health burden from exposure to combustion-derived particulate air pollution in domestic settings in Ireland and Scotland.

**Methods:**

The study focused on solid fuel combustion used for heating and the use of gas for cooking. PM_2.5_ (particulate matter with an aerodynamic diameter < 2.5 μm) was used as the pollutant mixture indicator. Measured PM_2.5_ concentrations in homes using solid fuels were adjusted for other sources of PM_2.5_ by subtracting PM_2.5_ concentrations in homes using gas for cooking but not solid fuel heating. Health burden was estimated for exposure indoors 6 pm - midnight, or all day (24-hour), by combining estimated attributable annual PM_2.5_ exposures with (i) selected epidemiological functions linking PM_2.5_ with mortality and morbidity (involving some re-scaling from PM_10_ to PM_2.5_, and adjustments ‘translating’ from concentrations to exposures) and (ii) on the current population exposed and background rates of morbidity and mortality.

**Results:**

PM_2.5_ concentrations in coal and wood burning homes were similar to homes using gas for cooking, used here as a baseline (mean 24-hr PM_2.5_ concentrations 8.6 μg/m^3^) and so health impacts were not calculated. Concentrations of PM_2.5_ in homes using peat were higher (24-hr mean 15.6 μg/m^3^); however, health impacts were calculated for the exposed population in Ireland only; the proportion exposed in Scotland was very small. The assessment for winter evening exposure (estimated annual average increase of 2.11 μg/m^3^ over baseline) estimated 21 additional annual cases of all-cause mortality, 55 of chronic bronchitis, and 30,100 and 38,000 annual lower respiratory symptom days (including cough) and restricted activity days respectively.

**Conclusion:**

New methods for estimating the potential health burden of combustion-generated pollution from solid fuels in Irish and Scottish homes are provided. The methodology involves several approximations and uncertainties but is consistent with a wider movement towards quantifying risks in PM_2.5_ irrespective of source. Results show an effect of indoor smoke from using peat (but not wood or coal) for heating and cooking; but they do not suggest that this is a major public health issue.

## Background

Electricity and gas are the main fuels used for heating and cooking in Ireland (throughout this paper, ‘Ireland’ and ‘Irish’ refer to the Republic of Ireland unless otherwise stated) and Scotland. In both countries however some homes still use solid fuel (coal, wood, and peat) as residential energy sources. These contribute to both indoor and outdoor air pollution and the health consequences of this are largely unexplored. The need to understand these consequences is increased because use of solid fuels is likely to change, influenced by policies to reduce Greenhouse Gas emissions which support greater use of renewable or biomass fuels like wood and reduced use of fossil fuels like coal. Peat has been considered a fossil fuel but its status as fossil or renewable fuel is unclear [1].

The Indoor Air Pollution and Health (IAPAH) project [2] aimed to quantify the levels of indoor air pollution (IAP) in Irish and Scottish homes using different types of combustion fuels (coal, wood and peat for heating and gas for cooking) and to estimate the health impacts of exposure to IAP generated from the combustion of these fuels; it also considered exposure to Environmental Tobacco Smoke (ETS) in homes. This manuscript reports work within IAPAH with the aim of estimating the population health burden from IAP exposure attributable to solid fuel use as a primary source of heating, and to using gas for cooking in domestic settings in Ireland and Scotland.

Scientific methods for estimating the health burden attributable to air pollution, and Health Impact Assessment (HIA) of pollution changes, have mainly focused on outdoor air pollution. There is no corresponding established methodology for HIA of indoor air pollution from indoor sources. In 2007, the Scientific Committee on Health and Environmental Risks [3] identified gaps in the scientific knowledge needed for a health-based risk assessment strategy on Indoor Air Quality (IAQ). Many of the gaps relate to the lack of specific information on source pollutant concentrations, exposure patterns and health effects of specific indoor air pollutants; IAPAH was designed to address these issues, with application to Ireland and Scotland.

There was limited directly relevant existing information. Much work has been published on indoor air pollutants and the burning of solid or biomass fuels for heating and cooking in developing countries [4,5] but data from such studies cannot be extrapolated reliably to more economically developed settings because of major differences in housing, ventilation, heating and cooking appliances, and fuels used. Some studies have investigated how the use of solid fuels for cooking or heating in the home contributes to IAP in developed countries, in homes that use wood [6-8], gas [9], coal [10,11] or peat [12], but available information is limited. As described elsewhere [13] and summarised below, the IAPAH study itself therefore included new measurements in Irish and Scottish homes, of which the data collected from 100 homes were used for analysis.

Air pollutants found in the indoor environment can play a significant role in human health. This is unsurprising, in that data from the USA and the EU indicate a significant proportion of our time is spent indoors [14], and vulnerable groups such as young children and the elderly can spend up to 100% of their time indoors [15]. Under conditions of very high exposures compared with Ireland and Scotland [4,5,13], exposure to IAP from solid fuel combustion has been linked to the development or exacerbation of chronic respiratory illnesses such as asthma, allergies and chronic obstructive pulmonary disease (COPD), and cardiovascular disease [4,5]. Ireland’s mortality rate from respiratory disease is over twice the EU average [16], while both Ireland and the United Kingdom have a particularly high prevalence of childhood allergy and asthma [17]. While it would be wrong to presume that IAP was a major cause of these differences, it is important to understand what role IAP may play.

To do this we developed and used a methodology based on assuming that, for policy purposes, the risks to health from IAP can be approximated by adapting concentration-response functions from outdoor air pollution, in the metric of PM_2.5_. One focus of the paper is to describe and discuss the strengths and weaknesses of this strategy; a second is to describe and discuss the substantive results because we think these are sufficiently reliable, at least qualitatively, to inform the development of policy. Methods and results are described in more detail in a series of longer reports [2].

Within IAPAH, the health burden attributable to ETS exposure has been estimated using both a method based on PM_2.5_ (pollutant-based approach) and another, source-based, approach, based on whether or not there is exposure to ETS in the home. As we discuss in this manuscript, we think that a source-based approach to health burden from IAP attributable to solid fuel use is not viable, and so a different approach, such as we adopted with PM_2.5_, is necessary if estimates are to be made.

## Methods

### The pollutant based approach to estimating health burden

Working jointly with the EU HEIMTSA project (http://www.heimtsa.eu), the ‘full chain’ approach to environmental HIA (http://www.integrated-assessment.eu/), developed and promoted by EU-funded projects such as ExternE (http://www.externe.info/externe_d7/), HEIMTSA and INTARESE (http://www.intarese.org), was adapted for application to IAP from combustion. This approach tracks the fate of pollutants from their source, through environments which allow for human exposure, to their specific health impacts. This requires considering as an integrated whole, the entire chain or pathway from pollution source through to health outcome, and managing the transitions between steps of the pathway; e.g. the exposure metric used for estimating exposures must be the same as the exposure metric used for estimating exposure-related risks to health.

The pollutant-based approach takes one or more signature pollutants as a marker of the entire combustion mixture from the source of interest. In line with HIA of outdoor air pollution, PM_2.5_ (particulate matter with an aerodynamic diameter of less than 2.5 μm) was used as the index of combustion mixtures for indoor sources.

**Figure 1:**
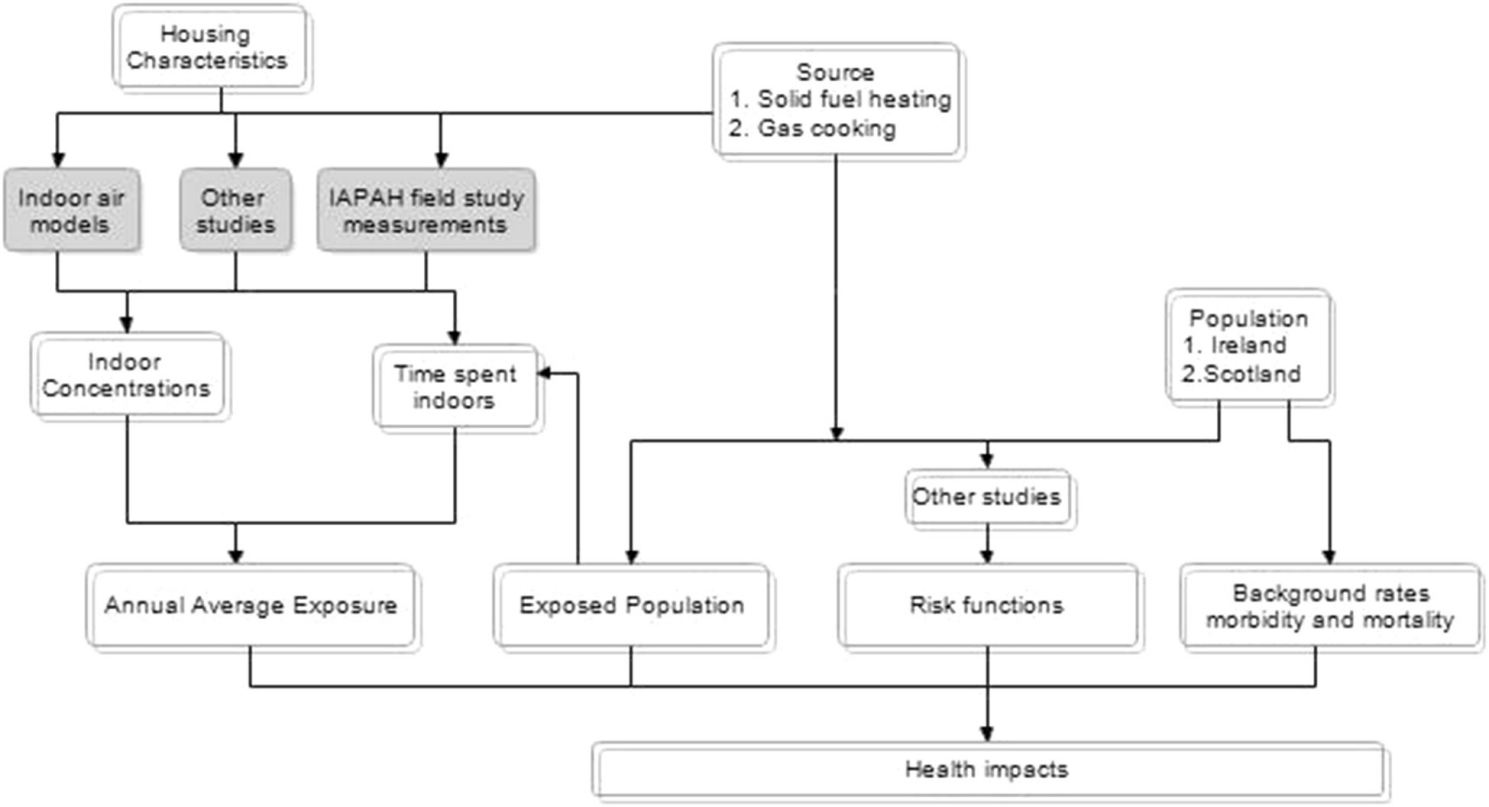
**Application of the pollutant**-**based approach within IAPAH** (**grey boxes are unique to the IAPAH study**)**.** Note: Indoor concentrations is the additional increment in concentrations due to using peat for heating, over and above the baseline of homes using gas for cooking but no solid fuel.

This strategy then proceeds to an assessment of health burden by combining information about (i) the relevant population exposed to IAP from combustion sources indoors; (ii) concentrations of relevant pollutants (i.e. PM_2.5_) within homes with combustion sources of pollution [13]; (iii) the risk to health of exposure indoors to those levels of PM_2.5_ and (iv) background rates of morbidity and mortality in the exposed population. To allow the assessment of health burden to be undertaken, information from multiple sources was required to provide the data required to populate each of the variables as described in Figure 1.

### Population exposure to solid fuel combustion / using gas for cooking

Total population data of Ireland for 2010 were obtained from the Eurostat statistics database [18]. The data file provided population figures by one year age group, sex and country. The total population of Ireland in 2010 was 4,467,854 (2,216,444 male, 2,251,410 female).

Total population estimates were obtained for Scotland for 2010 (mid-year estimates) from the General Register Office for Scotland. These estimates were also provided by sex and one-year age groups [19]. The total population of Scotland in 2010 was 5,222,100 (2,530,315 men and 2,691,785 females).

Information on the number of households using solid fuel in Ireland and Scotland is very limited. Most of the documents reviewed, e.g. [20,21] contained published general figures on solid fuel usage in reference to the total energy consumption, not on the number of households using specific solid fuels or the characteristics of those who use solid fuels. For a full list of sources consulted see [22].

Relevant information for Ireland was identified in the Irish Household Budget Survey (HBS) (most recent report available for our study, dated 2004/2005) [23], which is a survey of a representative random sample of all private households in Ireland. Data from this survey were analysed and summarised, giving detailed information on household population and the fuel used for heating and cooking, classified as gas, electric, oil and solid fuel, but not by type of solid fuel (coal, peat and wood). Data from Sustainable Energy Ireland [20] showed that the total residential energy used for coal (7.1%) and peat (9.3%) was similar as a percentage of all sources classified; a percentage for wood was not given; we assumed it was small compared to the use of coal as shown by the results on household income expenditure [23]. On that basis we assumed that the population using peat was approximately half of the population that used solid fuel, i.e. the same as coal and wood together [22].

In Scotland information on the number of households that use solid fuel for heating and cooking, and associated population characteristics, was obtained from the Scottish House Condition Surveys (SHCS) completed in 2005/06, 2007 and 2008. These surveys included approximately 3000 households per survey. Estimates of the percentage of the population living in households burning solid fuel for heating, or using gas for cooking, were calculated by the SHCS team (David McLaren, SHCS statistician, 2010; personal communication). Percentages on the population using solid fuel for heating and cooking were extracted directly from the survey and weighed for the total population. This included an estimate that <0.5% of the population in Scotland used peat for heating and cooking.

### Annual average PM_2.5_ concentrations

In order to estimate annual burden of disease attributable to the indoor combustion sources of interest, it was necessary to estimate annual average exposures to PM_2.5_ indoors, attributable to these combustion sources. This involved several linked stages.

Estimates of annual average concentrations were derived from a limited programme of measurements of indoor air pollution in homes in Ireland and Scotland from the IAPAH field study [13]. These included real-time measurements of PM_2.5_ over one approximately 24-hr period in each participating home; these were time-weighted to give a 24-hr value for each home.

For each source of interest, i.e. coal, wood and peat for heating and gas for cooking, it was intended to sample 10 homes in each of Scotland and Ireland that used that source only. In practice (Table 1) there was some variation in numbers achieved. Also, about 30% of the solid fuel homes sampled used solid fuels as secondary, rather than a primary heating fuel, but there were no statistically significant differences in concentrations between the primary and secondary heating homes and so all were retained in the analysis [22].

**Table 1:** **Concentrations of PM**_**2**.**5**_**in homes in Ireland and Scotland using coal**, **wood or peat as primary heating fuel**; **and numbers of homes sampled**

**Time**-**weighted 24**-**hour average PM**_**2**.**5**_**mean values (μg/m**^**3**^**)**	**Coal**	**Peat**	**Wood**	**Gas for cooking**
Ireland	8.4 (n = 12)	15.2 (n = 17)	4.8 (n = 5)	11.2 (n = 5)
(range)	(5–19)	(2–44)	(3–6)	(4–28)
Scotland	9.4 (n = 10)	18.0 (n = 3)	8.6 (n = 17)	7.4 (n = 11)
(range)	(1–17)	(8–34)	(2–23)	(2–13)
All	8.9 (n = 22)	15.6 (n = 20)	7.7 (n = 22)	8.6 (n = 16)
(range)	(1–19)	(2–44)	(2–23)	(2–28)

We were unable to obtain specific data on when the population as a whole was likely to be at home and exposed. Instead we considered two scenarios, one at home and exposed during evenings only (6pm until midnight) and the other, at home and exposed full-time, i.e. 24-hours. The available measurements were used to give estimates of average indoor concentrations of PM_2.5_ in non-smoking homes using various kinds of solid fuel for heating or using gas for cooking, for evenings and all day [13].

Next, in order to estimate PM_2.5_ attributable to solid fuel use, or gas for cooking, it was necessary to adjust these average concentrations of PM_2.5_ indoors for the effect of other sources, i.e. penetration indoors of outdoor air pollution, and all other indoor sources. Given that no measurements were available from an ideal control (i.e. electricity for cooking, no solid fuel combustion sources for heating and no ETS), several approaches to adjustment were considered, including estimation of both outdoor PM_2.5_ and indoor penetration [22]. The approach eventually adopted drew on a literature review of using gas for cooking, and of other indoor sources. The results suggested that the contribution to indoor PM_2.5_ mass concentrations from using gas for cooking was negligible over the 6- or 24-hr periods relevant to this study; i.e. PM_2.5_ in homes using gas from cooking could not be distinguished reliably from background [22]. Average PM_2.5_ concentrations in homes using gas for cooking were therefore taken as a ‘baseline’ for the contribution of all other sources, and were subtracted from average PM_2.5_ concentrations indoors in the homes using solid fuel to give PM_2.5_ concentrations attributable to using the solid fuel of interest; and we did not attempt to estimate a health burden from using gas for cooking.

Concentrations of PM_2.5_ in homes using coal and wood were very similar to those in homes using gas cooking [13], and so were very similar to background indoors from outdoor air and other sources; and therefore health impacts for burning coal and wood were also not estimated. However, the field study concentrations for peat (mean 24-hrs time weighted average 15.6 μg/m^3^) were higher than those for baseline / gas cooking (mean 24-hrs TWA PM_2.5_ concentrations 8.6 μg/m^3^) (Table 1). For both peat and gas cooking, the concentrations used were those from both Ireland and Scotland combined, so that these could be based on the greatest number of relevant measurements.

The decision was taken to estimate health impacts attributable to IAP from peat, but only in Ireland because, as described earlier, <0.5% of the population in Scotland was exposed. Finally, estimated annual average exposures were derived; assuming that exposure to PM_2.5_ indoors from peat burning was for 6 months of the year only.

### Health outcomes; risk functions; background rates; impact functions

By using conversion factors and by accepting approximations it was possible, in quantifying the health impacts of IAP, to use the extensive international research evidence linking PM from outdoor air pollution with a very wide range of health outcomes, rather than being restricted to using the much more limited evidence base linking IAP and health.

The functions selected for outdoor air were based largely on those used in in the HIA and Cost-Benefit Analysis (CBA) of the European Commission’s Clean Air for Europe (CAFÉ) programme [24,24]. The exceptions were the Concentration Response Functions (CRF) used for chronic bronchitis where an updated CRF based on the Swiss Cohort Study on Air Pollution and Lung and Heart Diseases in Adults (SAPALDIA) study [25] was used, and the CRF for respiratory hospital admissions which was based on a more recent meta-analysis from the INTARESE study [26]. Concentration response functions (CRFs) in the metric of PM_10_ were converted to CRFs in PM_2.5_ using a factor which represents, on average, the relationship between PM_10_ and PM_2.5_ in outdoor air across Europe. The conversion factor of 0.65 was obtained from concurrent measurements of PM_10_ and PM_2.5_ in the EU-wide Air Pollution and Health. An European Information System (Apheis-3) study [27] which reported city-specific conversion factors for 10 European cities, with six (Bordeaux, Gothenburg, Lille, Marseille, Stockholm, Toulouse) giving values 0.65-0.67; three (Athens, Madrid, Tel Aviv) giving values close to 0.5, and one (Cracow) giving a factor of 0.8. The selected functions in PM_10_ were ‘translated’ to PM_2.5_ using Equation 1.

(1) $$PM_{2.5}\mathrm{CRF}=\text{exp}(\ln\left(PM_{2.5}\mathrm{CRF}\right)=\exp\left(\frac{\ln\left(PM_{10}\mathrm{CRF}\right)}{0.65}\right)\;$$

The PM_2.5_ CRF can then be combined with the original background rate to obtain an impact function in the exposure metric of PM_2.5_[22].

Relationships between outdoor PM and health are based on PM as measured at background concentrations, at a distance from source and from most of the population at risk; whereas IAPAH is concerned with PM in the home from indoor combustion sources in the same room or nearby – this is more like PM measured as personal exposure rather than background concentrations. We decided therefore to ‘convert’ CRFs in annual average PM_2.5_ from outdoor air into equivalent exposure-response functions (ERFs) for outdoor air, and link these ERFs with annual average PM_2.5_ attributable to use of peat for heating. Conversion was based on annual averages because HIA of outdoor air pollution is based on annual averages. Annual average is the relevant time-period for CRFs based on longer-term exposure; and while CRFs for short-term exposure typically use 24-hr daily concentrations, when (as here) the CRFs are linear with no threshold, the aggregate daily impacts values over one year can also be estimated using annual average PM_2.5_[22]. To address this, a simple model was constructed of time spent in various micro-environments (indoors; outdoors in traffic; elsewhere outdoors) and associated average concentrations relative to background outdoors. A conversion or scaling factor was estimated as 0.7, by which the CRFs of outdoor air were divided to convert them to the required ERF [22]. The scaling factor is driven by the infiltration factor (0.55 (95% CI: 0.52-0.58) [22] and the time spent indoors. The at-risk population at various ages was then linked with estimated annual average exposures, with the ERFs, and with background rates, to give, for Ireland, the estimated annual burden of disease attributable to combustion of peat indoors. Background rates of disease were derived from a range of sources [22] including the Central Statistics Office, Ireland and the WHO Hospital Morbidity database.

Throughout, a simplifying convention was adopted as is usual when considering disease burden [28]. The calculations have been done as if the effect of exposure on disease and mortality were immediate; i.e. the effects of current exposure levels were estimated using current population and current background rates of morbidity and mortality, without taking account of any time lag between exposure and increased risk of disease or death.

## Results

### Population exposure to solid fuel combustion / using gas for cooking

Table 2 shows the percentage of population living in homes where solid fuel is used as primary fuel for heating, or gas is used for cooking, in Ireland and Scotland. Based on corresponding fuel usage data, it was assumed that the population in Ireland exposed to indoor pollution from peat as primary heating fuel is half of that reported as using any type of solid fuel, i.e. 4.2% of total households, with associated percentages of the population exposed, as given for solid fuel use in Table 2. This provides an estimate of 199,090 exposed individuals: 42,644 aged under 14 years, 22,568 aged 14–20, and 133,878 aged 21 years or more.

**Table 2:** Percentage of Irish and Scottish population living in households where solid fuel is used as primary heating fuel or gas for cooking

**Ireland**	**<14 years (%)**	**14**-**20 years (%)**	**Men**^**2 **^**21+ (%)**	**Women**^**2 **^**21+ (%)**	**Households sampled (%)**
Heating	9.5	11.8	8.5	9.3	8.4*
Gas Cooking^3^	23.7	22.2	26.0	25.3	26.0*
**Scotland**^**1**^	**<15 years (%)**	**15**-**25**^**2 **^**years (%)**	**Men**^**2 **^**>25 (%)**	**Women**^**2 **^**>25 (%)**	**Households sampled (%)**
Heating	1.0	1.5	1.9	1.6	2.5**
Gas Cooking^4^	57.5	53.3	54.9	53.8	49.3**

^1^ Scottish data for solid fuel use aggregate over coal, peat or wood, smokeless fuel, and anthracite.

^2^ The age-ranges used are unusual; we used slightly modified ranges to link with population numbers.

^3^ Gas cooking in Ireland: either piped gas or LPG.

^4^ Gas cooking in Scotland: i.e. gas cooker; or gas hob and electric oven;

*Percentage of a representative random sample of all private households in Ireland (total number sampled = 6,884).

** Percentage of households, total sample size of 9,194 for primary heating and 6,047 for cooking.

### Annual average concentrations

The mean 24-hr TWA concentration of PM_2.5_ attributable to peat was calculated as 7.1 μg/m^3^ (mean 24-hr average of 15.6 μg/m^3^[13] minus 8.5 μg/m^3^ (mean 24-hr average from cooking with gas homes as baseline)).

The annual average mean 24-hr attributable concentration to which residents were exposed was calculated as the estimated attributable concentration of 7.1 μg/m^3^ × 0.5 (to adjust for exposure occurring for approximately 6 ‘winter’ months of the year) giving an estimated annual average additional concentration of 3.55 μg/m^3^ based on 24-hr exposure and an estimated annual average additional concentration of 2.11 μg/m^3^ based on exposures in winter evenings only (6pm-midnight).

### Health outcomes; risk functions; background rates; impact functions

Table 3 summarises the health outcomes, risk functions, background rates and impact functions that were calculated and used in the HBA.

**Table 3:** **Summary of risk and impact functions used for each health outcome assessed for Ireland **[24]

**Health outcome**	**Age group**	**Background rate per 100**,**000**	**Risk function per 10 μg**/**m**^**3**^	**Risk function per μg**/**m**^**3 **^**personal exposure to PM**_**2**.**5**_	**Impact function: cases per 100**,**000 exposed persons per 10 μg**/**m**^**3 **^**personal exposure to PM**_**2**.**5**_
			**PM**_**10**_		
Chronic bronchitis	18+	390	22% [25]	5.11%	199
Cardiovascular hospital admissions	All ages	639	0.6% [2]	0.13%	8
Respiratory hospital admissions	All ages	1112	0.9% [26]	0.20%	22
Restricted activity days	18-64	2,200,000	4.75% [29]	0.68%	147,200
Lower respiratory symptom days (inc. cough)	5-14	5,600,000	4% [30]	0.89%	497,800
All-cause mortality	30+	1058	6% [31]	0.86%	91

### Health burden assessment

**Figure 2:**
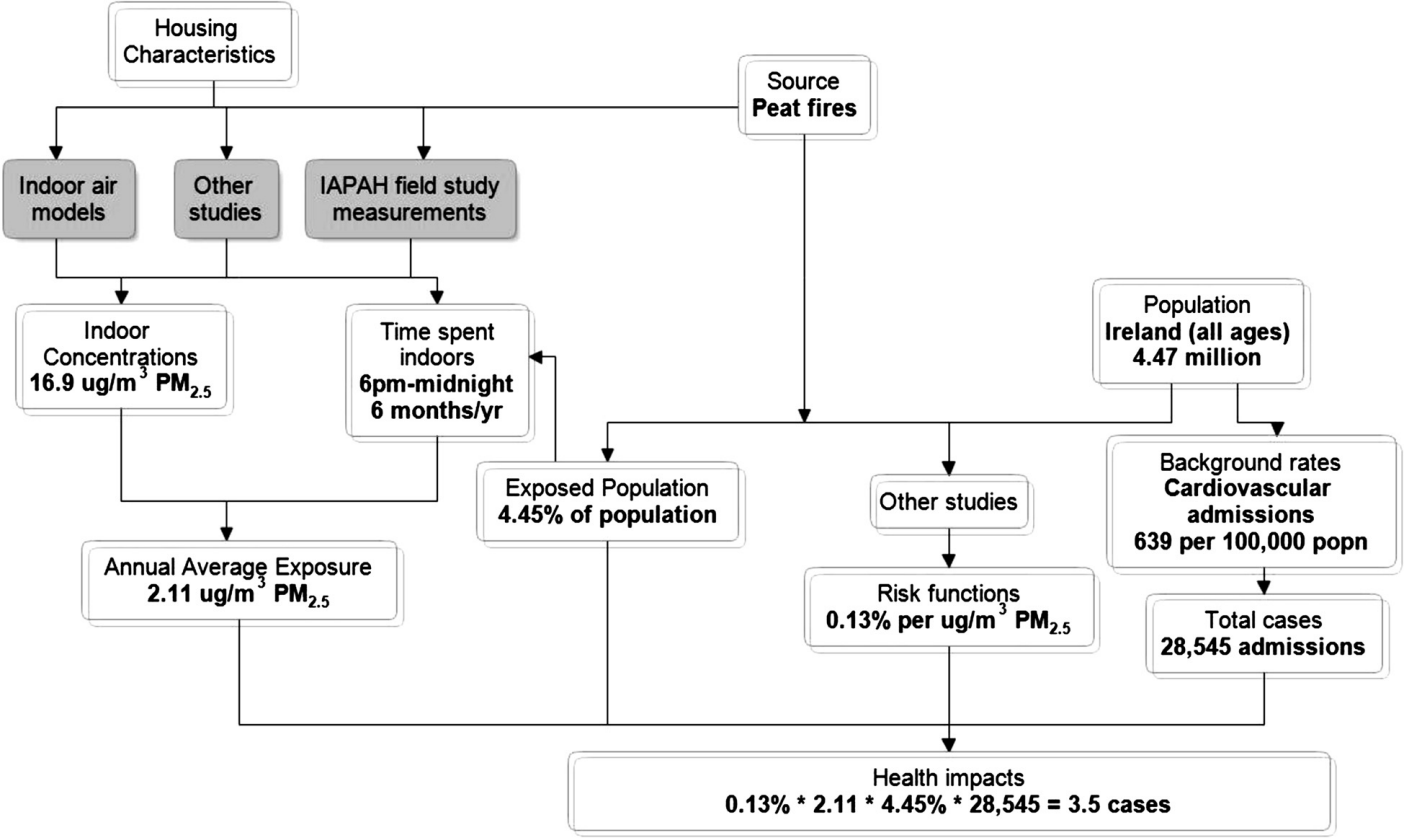
**Illustrative calculation of pollutant based approach - peat as a primary heating fuel in the evenings only**, **for 6 months of the year**, **in Ireland and hospital admissions for cardiovascular disease.** Note: Indoor concentrations is the additional increment in concentrations due to using peat for heating, over and above the baseline of homes using gas for cooking but no solid fuel.

An illustrative calculation, using as an example exposure to peat as a primary heating fuel in the evenings only, for 6 months of the year, in Ireland and hospital admissions for cardiovascular disease, is provided in Appendix 1 and summarised in Figure 2. (Note that there is some rounding in the presentation of the results).

The health impacts associated with peat-burning for heating in Ireland are shown in Table 4. Two exposure scenarios were considered: firstly based on exposures in winter evenings only, giving an annual average increase of 2.11 μg/m^3^; and secondly based on 24-hr exposure, in winter only, giving an annual average increase of 3.55 μg/m^3^. In summary, the assessment calculated 21 annual cases of all-cause mortality, 55 annual cases of chronic bronchitis, 4 annual cases of cardiovascular hospital admissions and 9 annual cases of respiratory hospital admissions attributable to an annual average increase in exposure of 2.11 μg/m^3^. Some 30,100 and 38,000 annual lower respiratory symptom days (including cough) and restricted activity days respectively were also estimated at these exposures. Results for all-day exposure were 1.68 times as large as those generated from evening exposures, reflecting higher evening concentrations (Table 4).

**Table 4:** Estimated annual burden on health in Ireland of indoor air pollution from burning peat as primary fuel in Ireland

				**Exposure winter evenings (6pm-midnight), concentration = 2.11 μg/m**^**3**^		**Exposure 24**-**hr**, **concentration** = **3**.**55 μg/m**^**3**^	
**Health endpoint**	**Age group**	**Total pop. at risk**	**% exposed**	**Annual no. cases**/**days**	**95% CI**	**Annual no**. **cases**/**days**	**95% CI**
Chronic bronchitis	18+	3,012,306	4.30	55^*^	(5–98)	91^*^	(8–163)
Cardiovascular hospital admissions	All ages	4,467,854	4.45	4^*^	(2–5)	6^*^	(3–9)
Respiratory hospital admissions	All ages	4,467,854	4.45	9^*^	(7–10)	15^*^	(12–17)
Restricted activity days	18-64	2,841,127	4.30	38,000^**^	(33,400-42,600)	63,300^**^	(55,600-71,100)
Lower respiratory symptom days (inc cough)	5-14	602,919	4.75	30,100^**^	(15,000-45,400)	50,200^**^	(25,000-75,700)
All-cause mortality	30+	2,559,015	4.20	21^*^	(7–38)	34^*^	(11–63)

^*^number of cases, ^**^ number of days.

## Discussion

This paper provides the first detailed estimates of the potential health burden of combustion-generated pollution from combustion of solid fuels at home for the Irish and Scottish populations. There were a number of methodological issues to overcome and we concentrate our discussion on these.

### Strength and limitations of the pollutant-based methodology used

There are strengths and limitations in using a pollutant-based approach compared with a source-based approach that uses a simple metric distinguishing between ‘exposed’ and ‘non-exposed’ people, as used e.g. in the earlier Global Burden of Disease (GBD) project [32]. One over-riding consideration is that the source-based approach was not viable in the present context, because (i) there is no substantial evidence base linking presence / absence of solid fuel use in Europe with chronic morbidity or mortality; and (ii) it is not meaningful to transfer, for use in Ireland and Scotland, relative risks from living in a house with solid fuel usage in developing countries, where many circumstances are different, including the extent of attributable IAP. When a source-based approach was used by GBD, no effects were quantified in Europe [32]. Although in this manuscript no effects were quantified for burning wood or coal for heating in Europe, the pollutant-based approach reached this position in a more informed way.

There are two other specific benefits. One is that the pollutant-based approach takes account of intensity of exposure, i.e. the concentrations actually experienced; and intuitively this is important, with the likelihood that risks are higher when exposures are higher, other things being equal. Secondly, while clearly there are issues (discussed below) in using risk functions in PM_2.5_ from outdoor air to quantify the effects of indoor air pollution, doing so allows quantification of a much wider range of health outcomes than would be possible from the much more limited evidence base linking indoor air pollution and health.

As indicated above, the main disadvantage of the pollutant-based approach is the approximation or uncertainty in using relationships in PM_2.5_ from outdoor air to quantify the health effects of PM_2.5_ from indoor air sources. PM is a complex mixture whose detailed characteristics depend on source, and different sources also produce different mixtures of co-pollutants – gases, and coarse particles. Either of these aspects could lead to differing health effects caused by different mixtures from IAP and outdoor air pollution, even though indexed by similar exposures to PM_2.5_. For example, PM_2.5_ in outdoor air typically contains a substantial proportion of secondary PM, whereas PM indoors attributable to solid fuel use indoors will consist entirely of primary combustion PM.

However similar considerations apply to outdoor air pollution at different places and times, where PM comes from different sources and with different mixtures of gases [33]. Despite some evidence and much speculation about consequent differences in toxicity, current standard practice with outdoor air pollution HIA is not to try to express any such differences quantitatively [34]. Clearly there are some additional uncertainties in applying the same assumptions to IAP but it was considered that this extrapolation is sufficiently similar to established practice for outdoor air that it is a reasonable way forward, especially given the lack of alternative methods. We were encouraged in this view by [35] who found a consistency in estimated risks of cause specific cardiopulmonary mortality from three very different sources of air pollution – outdoor air, ETS and active smoking – when expressed in the metric of log PM_2.5_ dose; suggesting that health effect relationships from exposure to PM_2.5_ are robust across differences in source, composition and intensity. This approach was further developed [36] and then supported by the most recent analyses of the internationally renowned GBD study [37], whose CRFs for air pollution are based on PM_2.5_ from these three diverse sources.

Relationships between outdoor PM and health outcomes are based on PM measured at background concentrations, at distance from source and from most of the population at risk, whereas this study is concerned with PM in the home from indoor combustion sources in the same room or nearby. To address this difference, a conversion or scaling factor was applied to convert the outdoor air CRFs to the ERFs needed for the assessment of health burden. The ease with which this can be done numerically (it’s a simple multiplication) should not hide that there are complex underlying issues which deserve further investigation. One is the variation in time and space between annual average concentration of PM_2.5_ and the distribution of personal exposures (to pollution from outdoor sources) in the local population – we used a simple model based on average concentrations and exposures, which does not attempt to take account of individual variation in exposure or susceptibility.

While there are issues about the reliability of such a conversion factor, a more fundamental point is whether such a conversion should be done at all. The IAPAH field study measurements [13] do not exactly match either the situation outdoors or personal exposures. On balance, they were considered more similar to personal exposures and so the conversion was applied. This understanding of the relationship between static micro-environment based measures and personal exposure is one particular aspect that warrants wider discussion and consideration.

The present report focuses on burden attributable to current levels and exposure to IAP from peat burning in Ireland; it is not an analysis of the health effects of a change in policy and practice. Such an application would also require consideration of the time-lag (‘cessation lag’) between changes in exposure and changes in consequent health impacts. This is an aspect which needs to be considered in the CBA of potential policy changes but which typically is overlooked or ‘fudged’ in estimates of burden as is done here [28]; GBD, for example [37], bypasses the issue.

The effect on PM_2.5_ concentrations of burning gas for cooking was estimated as being very small. Consequently the health effects in the metric of PM_2.5_ from using gas for cooking could not be estimated. NO_2_ could have been used as an alternative indicator pollutant: using gas for cooking is widely linked with increases in NO_2_ indoors and there are relationships linking NO_2_ in outdoor air with a wide range of health outcomes, including mortality. However, these are widely understood as reflecting primarily an effect of the complex mixture, including PM, from traffic combustion, rather than an effect of NO_2_ per se. It was considered that these could not be transferred with confidence from outdoor to indoor air, and so quantification using NO_2_ as a marker was not attempted. Use of functions in NO_2_ from studies of IAP (see e.g. [38]) would have led to a much narrower range of health outcomes than is possible with PM_2.5_. It would be wrong however to conclude, on the basis of low attributable PM_2.5_ concentrations, that using gas for cooking has no adverse effects on health.

### Issues in accessing relevant data

As noted in Figures 1 and 2, HIA in this context involves estimating and integrating information from a wide variety of sources.

IAPAH aimed to estimate the health burden on the populations of Ireland and Scotland, attributable to (i) burning coal for heating; (ii) burning wood for heating; (iii) burning peat for heating; and (iv) using gas for cooking. This gave four populations to estimate in each of two countries; and these populations needed to be disaggregated by age and, to some extent, by gender, for compatibility with the CRFs / ERFs used elsewhere in the analysis. There were no immediate sources of relevant data, and insofar as information was available, it was not disaggregated by fuel type to the extent required. Consequently, considerable work was needed to identify, collate and cross-reference across multiple sources, each of which gave a partial insight, in order to estimate the exposed populations at the required degree of granularity. Both the limited information available from surveys in both countries, and the wider information on fuel usage (but not on corresponding population exposed) were needed.

The IAPAH field study [13] proved essential from the viewpoint of estimating PM_2.5_ attributable to source. It had however, what appeared to be a limitation: it did not have a designed-in ‘control’ set of homes (e.g. using electricity for heating and cooking) which would allow adjustment for other sources of PM_2.5_ indoors, such as PM_2.5_ from cooking, from re-suspension of settled dust as people got on with living their lives at home, or from penetration indoors of outdoor PM_2.5_. However, when it became clear that the measurements of PM_2.5_ in homes using gas for cooking could not be distinguished reliably from the aggregate of these corrections, and when limited literature review reinforced that the contribution to PM_2.5_ from gas flames during cooking would be very small, it was possible to use homes using gas for cooking as a baseline for all other sources when estimating PM_2.5_ due to solid fuel use.

We did not know of any existing source that would give us time-activity patterns in the detail required (e.g. vacuum cleaning, walking); nor did we wish to extrapolate from the limited time-activity data collected in the IAPAH field studies [13]. Instead the assumption of two different scenarios was used, one of exposure during evenings only, the other with exposure all day. Neither are fully realistic but together they provide some reasonable estimates of the range of potential population exposures and consequent health burden. We acknowledge that it is difficult to be certain of the representativeness of our study sample in the field study and make no claim that the single 24 hour measurement collected in 20 homes for peat burning and 16 homes for cooking with gas are representative of the concentrations experienced in all such homes in Ireland. The CRFs used from studies of outdoor air are standard ones. There are of course uncertainties, to some extent reflected in the 95% CIs. Information on background rates varied in quality according to the health outcome, for example, there is a greater likelihood of having routinely available data for more extreme events. Considering jointly the uncertainties in risk estimates and background rates, it is considered reasonable to put greatest confidence in results for mortality and for hospital admissions, with much greater uncertainties in results for chronic bronchitis, restricted activity days and days reporting respiratory symptoms.

### Substantive results

No estimates were made of the health burden attributable to the particulate air pollution from combustion of gas for cooking or for the combustion of coal and wood for heating. This should not be interpreted as saying that there are no adverse health effects; for example, a meta-analysis in the metric of NO_2_ suggests that use of a gas cooker at home increases the risk of children’s respiratory illness [38]. It is however reasonable to infer that any associated burden of disease is small, in terms of overall public health in Ireland and in Scotland, and is unlikely to be associated with mass concentrations of fine particulate aerosol.

For the combustion of peat for heating, a contribution to indoor concentrations of PM_2.5_ was identified, though PM_2.5_ concentrations from all sources within homes burning peat was of the same order as concentrations of PM_2.5_ outdoors in many cities. This is, of course, higher than outdoor PM_2.5_ concentrations in the largely rural areas of Ireland and Scotland where peat is used for heating. The estimated population exposed in Scotland was so small that, given that the attributable concentrations of PM_2.5_ were not large, a HBA was not attempted. An assessment of health burden was undertaken for peat burning in Ireland and the resulting estimates show, as expected, some limited impacts on serious health outcomes, including mortality; and more numerous impacts on mild or transient conditions such as lower respiratory symptom days.

## Conclusions

It is difficult to assess the accuracy of the methodology as a whole as the approach is new. As such, further methodological development is needed. It involves a key underlying methodological assumption, that relationships in the metric of PM_2.5_ from outdoor air epidemiology can be adapted for use in quantifying the health impacts of air pollution from combustion sources indoors. This needs to be discussed more widely and a consensus reached in the wider research community on its strengths and weaknesses and its range of legitimate application. The recent use by the GBD project of quantification in PM_2.5_ unified across very different sources [37] should highlight the issue considerably.

While the fundamental assumptions will understandably gain attention, a very important issue practically is that of obtaining the necessary information to implement the approach. In the present study some quite complex processing and linking of data from various sources was needed to estimate both the population exposed to IAP from using various kinds of solid fuels for heating, and the background rates of morbidity in the non-exposed population. We under-estimated these difficulties and we encourage others to learn from that, as we also hope to do.

One of the advantages of this study, despite all its uncertainties, is the clear illustration of the data gaps. Further work to fill these gaps not only on population exposed and background morbidity but also on more indoor- specific exposure response functions and greater understanding of the relationship between exposure to different sources, personal exposure and health is recommended.

In general, the indoor combustion sources examined had little impact on concentrations of PM_2.5_ indoors. Use of peat for heating in Ireland was the only source where health impacts were estimated, and these estimates were found to be small – risks are higher in individual homes using peat for heating, although not higher than those experienced from exposure to outdoor air pollution in many cities in Western Europe; and the number of people exposed is not large.

Consequently, this study does not suggest that exposures in homes in Ireland and Scotland to IAP from using solid fuel for heating (and cooking), is a major public health issue. However, to inform policy development more fully, it would be useful to estimate also the effect on outdoor air pollution, and the resulting health impacts, from burning solid fuels in homes in Ireland and Scotland.

## Appendix

### Appendix 1: Illustrative calculation

Peat used as a primary heating fuel in the evenings only, for 6 months of the year, in Ireland and hospital admissions for cardiovascular disease.

Step 1: Background rates

○ The overall background rate of cardiovascular hospital admissions for Ireland is 638.9 per 100,000.

○ This background rate is applied to the 2010 population (all ages) to obtain the number of cardiovascular admissions, annually, in the 2010 population.

■ (638.9 × 4,467,854)/100,000 = 28,545 admissions.

○ The percentage exposed to peat in each age group is derived from Table 3 and earlier.

■ 4.45% exposed (in total population).

○ The number of cardiovascular admissions in the total population (28,545) is then multiplied by the proportion exposed (4.45%) to obtain the number of cardiovascular admissions in the exposed population.

■ 1,270 admissions.

Step 2: Relative risk

○ The relative risk (RR) per 10 μg/m^3^ PM_10_ for cardiovascular hospital admissions is 1.006 (95% CI: 1.003-1.009).

○ The RR per 10 μg/m^3^ PM_10_ is converted to an RR per 10 μg/m^3^ PM_2.5_ which results in a RR = 1.009 change in admissions per 10 μg/m^3^ PM_2.5_.

○ The RR in the exposure metric of PM_2.5_ is then adjusted to personal exposure by dividing the excess risk (0.009) by 0.7 = 0.013.

■ RR = 1.013 change in admissions per 10 ug/m3 PM_2.5_ personal exposure.

○ The RR for personal exposure is then divided by 10 to obtain the % change in personal exposure per μg/m^3^ PM_2.5_.

● 0.13% per μg/m^3^ PM_2.5_ personal exposure.

● (The same is done for the upper and lower 95% CIs).

Step 3: Health burden

○ The annual average PM_2.5_exposure indoors, based on evenings spent at home is then used with the background rate in the exposed population and the RR for personal exposure to obtain the burden of cardiovascular hospital admissions due to peat exposure.

■ 0.13% × 2.11 μg/m^3^ PM_2.5_ × 1,270 cases among exposed population = 4 attributable cases.

## Abbreviations

APHEIS: Air pollution and health. An European information system; CAFÉ: European commission’s clean air for Europe; CBA: Cost-benefit analysis; COPD: Chronic obstructive pulmonary disease; CRF: Concentration response functions; ETS: Environmental tobacco smoke; ERFs: Exposure-response functions; ExternE: External costs of energy; GBD: Global burden of disease; HEIMTSA: Health and environment integrated methodology and toolbox for scenario assessment; HIA: Health impact assessment; IAPAH: Indoor air pollution and health; IAP: Indoor air pollution; IAQ: Indoor air quality; INTERASA: Integrated assessment of health risk of environmental stressors in europe; PMx: Particulate matter with an aerodynamic diameter < x μm; SHCS: Scottish house condition surveys; SAPALDIA: Swiss cohort study on air pollution and lung and heart diseases in adults; WHO: World health organization.

## Competing interests

The author(s) declare that they have no competing interests.

## Authors’ contributions

KG was involved in gaining funding for the study, overall study design and interpretation of the data. KG led the drafting of the manuscript. FH was involved in gaining funding for the study, overall study design and interpretation of the data and led the response to the reviewers’ comments. HC contributed to the identification of relevant data for the HIA and statistical analysis of the study. AS and ASJ contributed to the estimation, analysis and identification of relevant data for the HIA. SS and JA was involved in gaining funding for the study, overall study design and discussions relating to the use of exposure data. MC was involved in gaining funding for the study, overall study design and discussions relating to the use of exposure data. MC was also the project Principal Investigator. All authors assisted with the drafting and revision of the manuscript and have read and approved the final version.

## References

[B1] World Energy Council Survey of Energy Resources 2007 http://www.worldenergy.org/publications/survey_of_energy_resources_2007/peat/704.asp

[B2] Coggins MA Semple S Hurley F Shafrir A Galea KS Cowie H Sanchez-Jimenez A Garden C Whelan P Ayres JG Indoor Air Pollution and Health (IAPAH). STRIVE Report (2008-EH-MS-8-S3) Dublin: Environmental Protection Agency http://www.epa.ie/pubs/reports/research/health/iapahreportmcoggins.html

[B3] European Commission Scientific Committee on Health and Environmental Risks (SCHER) "Opinion on risk assessment on indoor air quality" DG Health and Consumers of the European Commission 2008 Brussels: European Commission http://ec.europa.eu/health/ph_risk/committees/04_scher/docs/scher_o_055.pdf

[B4] Kurmi OP Gaihre S Semple S Ayres JG Acute exposure to biomass smoke causes oxygen desaturation in adult women Thorax 2010 65 221 228 10.1136/thx.2009.124644 20880877

[B5] Fullerton DG Semple S Kalambo F Suseno A Malamba R Henderson G Ayres JG Gordon SB Biomass fuel use and indoor air pollution in homes in Malawi OEM 2009 66 777 783 10.1136/oem.2008.045013 PMC2760244 19671533

[B6] Levesque B Allaire S Gauvin D Koutrakis P Gingras S Rhainds M Prud’Homme H Duchesne JF Wood Burning appliances and indoor air quality Sci Total Environ 2001 281 47 62 10.1016/S0048-9697(01)00834-8 11778959

[B7] Fine PM Cass GR Simoneit BR Chemical characterzation of fine particle emissions from the fireplace combustion of woods grown in the Southern United States Environ Sci Technol 2002 36 1442 1451 10.1021/es0108988 11999049

[B8] Gustafson P Ostman C Sallsten G Indoor levels of polycyclic aromatic hydrocarbons in homes with or without wood burning for heating Environ Sci Technol 2008 42 5074 5080 10.1021/es800304y 18754350

[B9] Garcia Algar O Pichini S Basagana X Puig C Vall O Torrent M Harris J Sunyer J Cullinan P Concentrations and determinants of NO_2_ in homes of Ashford, UK and Barcelona and Menorca, Spain Indoor Air 2004 14 298 304 10.1111/j.1600-0668.2004.00256.x 15217483

[B10] Moriske HJ Drews M Ebert G Menk G Scheller C Schondube M Konieczny L Indoor air pollution by different heating systems: coal burning, open fireplace and central heating Toxicol Lett 1996 88 349 354 10.1016/0378-4274(96)03760-5 8920759

[B11] Henderson KA Parry S Matthews IP Real-time measurement of short-term peaks in environmental CO concentrations in the homes of the elderly in South Wales JESEE 2006 16 525 530 10.1038/sj.jes.7500491 16721412

[B12] Guo L Lewis JO McLaughlin JP Emissions from Irish domestic fireplaces and their impact on indoor air quality when used as a supplementary heating source Global NEST J 2008 10 209 216

[B13] Semple S Garden C Coggins M Galea KS Whelan P Cowie H Sánchez-Jiménez A Thorne PS Hurley JF Ayres JG Contribution of solid fuel, gas combustion, or tobacco smoke to indoor air pollutant concentrations in Irish and Scottish homes Indoor Air 2012 22 212 223 10.1111/j.1600-0668.2011.00755.x 22007695 PMC3573694

[B14] Kleipeis NE Nelson WC Ott WR Robinson JP Tsang AM Switzer P Behar JV Hern SC Engelmann WH The National Human Activity Pattern Survey (NHAPS): a resource for assessing exposure to environmental pollutants JESEE 2001 11 231 252 10.1038/sj.jea.7500165 11477521

[B15] Bonnefoy XR Annesi-Maesona I Aznar LM Braubachi M Croxford B Davidson M Ezratty V Fredouille J Ganzalez-Gross M Van Kamp I Maschke C Mesbah M Moisonnier B Monolbaev K Moore R Nicol S Niemann H Nygren C Ormandy D Robbel N Rudnai P Review of evidence on housing and health Proccedings of the 4th Ministerial Conference on Environment and Health 2004 Hungary, Budapest

[B16] Brennan N McCormack S O'Connor T Ireland Needs Healthier Airways and Lungs – the Evidence (INHALE) 2008 2 Irish Thoracic Society: Dublin 10.1183/09031936.04.00025604 15293624

[B17] ISAAC International Study of Asthma and Allergies in childhoodhttp://isaac.auckland.ac.nz/10.1002/ppul.20525 17123321

[B18] European Commission EuroStat: Your key to European Statistics Total population databasehttp://epp.eurostat.ec.europa.eu/portal/page/portal/eurostat/home/

[B19] General Registry Office for Scotland Mid 2010 population estimates for Scotland http://www.gro-scotland.gov.uk/statistics/theme/population/estimates/mid-year/2010/index.html

[B20] Energy in Ireland 1990–2007 http://www.seai.ie/Publications/Statistics_Publications/Energy_in_Ireland/Energy_in_Ireland_1990-2007.pdf21481289

[B21] Scottish Energy Study http://www.scotland.gov.uk/Publications/2006/01/19092748/5

[B22] Hurley F Shafrir A Cowie H Sánchez Jiménez A Ayres JG Coggins M Semple S Galea KS Indoor Air and Health in Ireland and Scotland (IAPAH) Supplementary Report 3: Estimation of the Health Burden due to Solid Fuel Use and Use of Gas for Cooking http://erc.epa.ie/safer/downloadCheck.jsp?isoID=282&rID=10434&atID=3315

[B23] Central Statistics Office Ireland Irish Household Budget Survey 2004/2005 http://www.cso.ie/en/media/csoie/releasespublications/documents/housing/hbsfinal/webcomplete.pdf

[B24] Hurley F Hunt A Cowie H Holland M Miller B Pye S Watkiss P Methodology for the Cost-Benefit Analysis for CAFÉ: Volume 2: Health Impact Assessment 2005 European Commission http://ec.europa.eu/environment/archives/cafe/pdf/cba_methodology_vol2.pdf

[B25] Schindler C Keidel D Gerbase MW Zemp E Bettschart R Brändli O Brutsche MH Burdet L Karrer W Knöpfli B Pons M Rapp R Bayer-Oglesby L Künzli N Schwartz J Liu LJ Ackermann-Liebrich U Rochat T SAPALDIA Team Improvements in PM10 Exposure and Reduced Rates of respiratory symptoms in a cohort of Swiss adults (SAPALDIA) Am J Respir Crit Care Med 2009 179 579 587 10.1164/rccm.200803-388OC 19151198

[B26] Hoek G Boogaard H Knol A de Hartog J Slottje P Ayres JG Borm P Brunekreef B Donaldson K Forastiere F Holgate S Kreyling WG Nemery B Pekkanen J Stone VW van der Sluijs J Concentration response functions for ultrafine particles and all-cause mortality and hospital admissions: results of a European expert panel elicitation Environ Sci Technol 2010 44 476 482 10.1021/es9021393 19958027

[B27] Boldo E Medina S Le Tertre A Hurley F Mucke HG Ballester F Aguilera I Eilstein D Apheis: Health Impact Assessment of Long-term Exposure to PM(2.5) in 23 European Cities Eur J Epidemiol 2006 21 449 458 10.1007/s10654-006-9014-0 16826453

[B28] COMEAP The mortality effects of long term exposure to particulate air pollution in the United Kingdom 2010 Health Protection Agency for the Committee on the Medical Effects of Air Pollutants http://www.hpa.org.uk/webc/HPAwebFile/HPAweb_C/1317137012567

[B29] Ostro BD Air pollution and morbidity revisited: A specification test J Environ Econ Manage 1987 14 87 98 10.1016/0095-0696(87)90008-8

[B30] Ward DJ Ayres JG Particulate air pollution and panel studies in children: a systematic review Occup Environ Med 2004 61 13 10.1136/oem.2003.007088 PMC1740745 15031404

[B31] Pope CA III Burnett RT Thun MJ Calle EE Krewski D Ito K Thurston GD Lung cancer, cardiopulmonary mortality, and long-term exposure to fine particulate air pollution JAMA 2002 287 1132 1141 10.1001/jama.287.9.1132 11879110 PMC4037163

[B32] Smith KR Mehta S Maeusezahl-Feuz M Ezzati M, Lopez AD, Rodgers A, Murray CLP Indoor air pollution from household use of solid fuels Comparative Quantification of Health Risks 2004 Geneva: World Health Organization 1435 1493

[B33] Shafrir A Sánchez Jiménez A Hurley F Cowie H Galea KS Indoor Air and Health in Ireland and Scotland (IAPAH) Supplementary Report 1: Health Impact Assessment: General Methodology and Its Application to the IAPAH study http://erc.epa.ie/safer/downloadCheck.jsp?isoID=282&rID=10434&atID=3313

[B34] WHO Health relevance of particulate matter from various sources: Report on a WHO Workshop. Bonn, Germany 26–27 March 2007 2007 Copenhagen: World Health Organization

[B35] Pope CAI Burnett RT Krewski D Jerrett M Shi Y Calle EE Thun MJ Cardiovascular mortality and exposure to airborne fine particulate matter and cigarette smoke. Shape of the exposure-response relationship Circulation 2009 120 941 948 10.1161/CIRCULATIONAHA.109.857888 19720932

[B36] Pope CAI Burnett RT Turner MC Cohen A Krewski D Jerrett M Gapstur SM Thun MJ Lung cancer and cardiovascular disease mortality associated with ambient air pollution and cigarette smoke: Shape of the exposure-response relationships Environ Health Perspect 2011 119 1616 1621 10.1289/ehp.1103639 21768054 PMC3226505

[B37] Lim SS Vos T Flaxman AD Danaei G Shibuya K Adair-Rohani H Amann M Anderson HR Andrews KG Aryee M A comparative risk assessment of burden of disease and injury attributable to 67 risk factors and risk factor clusters in 21 regions, 1990–2010: a systematic analysis for the Global Burden of Disease Study 2010 The Lancet 2012 380 2224 2260 10.1016/S0140-6736(12)61766-8 PMC4156511 23245609

[B38] Hasselblad V Eddy DM Kotchmar DJ Synthesis of environmental evidence: nitrogen dioxide epidemiology studies J Air Waste Manage Assoc 1992 42 662 671 10.1080/10473289.1992.10467018 1627322

